# Supplementary material for: Difficulty in artificial word learning impacts targeted memory reactivation and its underlying neural signatures
Source: eLife. 2024 Nov 4;12:RP90930. doi: 10.7554/eLife.90930 (PMC11534334; doi:10.7554/eLife.90930)
Supplement: Supplementary file 1. [file elife-90930-supp1.docx]

**Supplementary table S1** List of the high-PP words and their phoneme and biphone probabilities

|  | ***G1 (High-PP)*** | ***Phonotactic Probabilities*** | | ***G2 (High-PP)*** | ***Phonotactic Probabilities*** | |
| --- | --- | --- | --- | --- | --- | --- |
| ***Number*** | ***Word*** | ***Phoneme*** | ***Biphone*** | ***Word*** | ***Phoneme*** | ***Biphone*** |
| 1 | baos | 0.180 | 0.005 | baqy | 0.126 | 0.004 |
| 2 | beon | 0.146 | 0.003 | basy | 0.205 | 0.006 |
| 3 | biox | 0.181 | 0.003 | bavo | 0.156 | 0.005 |
| 4 | biuq | 0.098 | 0.002 | beto | 0.167 | 0.006 |
| 5 | cayr | 0.114 | 0.000 | bevo | 0.125 | 0.004 |
| 6 | ceot | 0.145 | 0.002 | bisu | 0.173 | 0.004 |
| 7 | ceov | 0.070 | 0.001 | bitu | 0.160 | 0.005 |
| 8 | ceox | 0.135 | 0.000 | bixy | 0.121 | 0.003 |
| 9 | cion | 0.105 | 0.002 | catu | 0.145 | 0.004 |
| 10 | cioz | 0.070 | 0.001 | cipu | 0.088 | 0.002 |
| 11 | daon | 0.178 | 0.004 | devo | 0.126 | 0.004 |
| 12 | daot | 0.220 | 0.004 | disy | 0.177 | 0.005 |
| 13 | deop | 0.136 | 0.002 | fasu | 0.197 | 0.005 |
| 14 | deus | 0.146 | 0.002 | fato | 0.194 | 0.006 |
| 15 | diov | 0.117 | 0.004 | faxo | 0.152 | 0.002 |
| 16 | faop | 0.162 | 0.002 | gazu | 0.118 | 0.003 |
| 17 | faoq | 0.126 | 0.002 | gexo | 0.101 | 0.002 |
| 18 | faor | 0.169 | 0.007 | giny | 0.168 | 0.006 |
| 19 | faot | 0.215 | 0.004 | haqy | 0.114 | 0.004 |
| 20 | faox | 0.206 | 0.002 | haso | 0.200 | 0.007 |
| 21 | faoz | 0.138 | 0.002 | heso | 0.168 | 0.005 |
| 22 | gaor | 0.148 | 0.007 | hesu | 0.159 | 0.004 |
| 23 | geon | 0.121 | 0.003 | hevo | 0.113 | 0.003 |
| 24 | giur | 0.115 | 0.000 | jaqo | 0.081 | 0.000 |
| 25 | gius | 0.123 | 0.001 | jiry | 0.124 | 0.000 |
| 26 | giut | 0.162 | 0.002 | kapy | 0.204 | 0.021 |
| 27 | haor | 0.161 | 0.008 | kexu | 0.158 | 0.002 |
| 28 | heoq | 0.087 | 0.001 | kipo | 0.183 | 0.004 |
| 29 | heox | 0.167 | 0.001 | kipu | 0.173 | 0.002 |
| 30 | hion | 0.137 | 0.003 | kiqo | 0.146 | 0.001 |
| 31 | jaoq | 0.079 | 0.000 | kizo | 0.166 | 0.002 |
| 32 | jios | 0.101 | 0.002 | leqo | 0.084 | 0.002 |
| 33 | kaon | 0.219 | 0.018 | lexu | 0.099 | 0.002 |
| 34 | kaot | 0.261 | 0.018 | liwo | 0.092 | 0.003 |
| 35 | kaov | 0.187 | 0.017 | manu | 0.225 | 0.019 |
| 36 | kiuz | 0.151 | 0.001 | mawo | 0.144 | 0.006 |
| 37 | leon | 0.129 | 0.003 | meqy | 0.101 | 0.003 |
| 38 | leoq | 0.082 | 0.002 | mino | 0.206 | 0.006 |
| 39 | liux | 0.160 | 0.003 | mipy | 0.140 | 0.005 |
| 40 | miux | 0.184 | 0.002 | mivy | 0.127 | 0.004 |
