# Supplementary material for: Difficulty in artificial word learning impacts targeted memory reactivation and its underlying neural signatures
Source: eLife. 2024 Nov 4;12:RP90930. doi: 10.7554/eLife.90930 (PMC11534334; doi:10.7554/eLife.90930)
Supplement: Supplementary file 2. [file elife-90930-supp2.docx]

**Supplementary table S2** List of the low-PP words and their phoneme and biphone probabilities

|  | ***G3 (Low-PP)*** | ***Phonotactic Probabilities*** | | ***G4 (Low-PP)*** | ***Phonotactic Probabilities*** | |
| --- | --- | --- | --- | --- | --- | --- |
| ***Number*** | ***Word*** | ***Phoneme*** | ***Biphone*** | ***Word*** | ***Phoneme*** | ***Biphone*** |
| 1 | abpu | 0.069 | 0.002 | afut | 0.121 | 0.002 |
| 2 | abwo | 0.047 | 0.002 | afux | 0.112 | 0.000 |
| 3 | adro | 0.120 | 0.003 | agyv | 0.035 | 0.000 |
| 4 | adwo | 0.047 | 0.000 | ahon | 0.078 | 0.001 |
| 5 | adzo | 0.062 | 0.001 | ahun | 0.074 | 0.001 |
| 6 | afwo | 0.043 | 0.000 | ahuz | 0.039 | 0.001 |
| 7 | afzo | 0.058 | 0.000 | ajot | 0.120 | 0.002 |
| 8 | agno | 0.135 | 0.002 | alor | 0.118 | 0.008 |
| 9 | agvy | 0.055 | 0.000 | ebon | 0.078 | 0.002 |
| 10 | agzo | 0.059 | 0.002 | ebox | 0.111 | 0.001 |
| 11 | ahvy | 0.050 | 0.000 | ecyn | 0.070 | 0.000 |
| 12 | akvu | 0.074 | 0.001 | efon | 0.074 | 0.002 |
| 13 | alpo | 0.115 | 0.002 | ehos | 0.073 | 0.001 |
| 14 | alzu | 0.088 | 0.000 | ehyq | 0.007 | 0.000 |
| 15 | ampy | 0.083 | 0.009 | ehyt | 0.096 | 0.000 |
| 16 | ebry | 0.105 | 0.001 | ehyv | 0.022 | 0.000 |
| 17 | ebvy | 0.050 | 0.001 | ejyz | 0.019 | 0.000 |
| 18 | ecpy | 0.072 | 0.002 | ekoz | 0.062 | 0.002 |
| 19 | ecto | 0.107 | 0.003 | ekyr | 0.077 | 0.001 |
| 20 | edty | 0.092 | 0.000 | ekyz | 0.046 | 0.001 |
| 21 | edxu | 0.048 | 0.001 | elon | 0.114 | 0.005 |
| 22 | edxy | 0.051 | 0.001 | elyp | 0.088 | 0.000 |
| 23 | efny | 0.119 | 0.003 | elys | 0.102 | 0.000 |
| 24 | efqy | 0.023 | 0.000 | emos | 0.092 | 0.002 |
| 25 | efsy | 0.102 | 0.000 | emyn | 0.073 | 0.001 |
| 26 | efwy | 0.028 | 0.000 | ibon | 0.077 | 0.002 |
| 27 | egnu | 0.117 | 0.001 | icon | 0.085 | 0.001 |
| 28 | egro | 0.109 | 0.004 | icuq | 0.035 | 0.000 |
| 29 | egxy | 0.048 | 0.000 | idon | 0.077 | 0.002 |
| 30 | ekpo | 0.089 | 0.002 | idox | 0.110 | 0.000 |
| 31 | ibry | 0.104 | 0.000 | idoz | 0.043 | 0.001 |
| 32 | ibvu | 0.047 | 0.000 | idur | 0.069 | 0.000 |
| 33 | idno | 0.129 | 0.001 | ihos | 0.072 | 0.001 |
| 34 | idqo | 0.033 | 0.000 | ijut | 0.108 | 0.002 |
| 35 | igpy | 0.060 | 0.002 | ijyv | 0.021 | 0.000 |
| 36 | igru | 0.098 | 0.002 | iluq | 0.063 | 0.002 |
| 37 | igty | 0.089 | 0.000 | ilyt | 0.140 | 0.000 |
| 38 | igvo | 0.053 | 0.001 | ilyx | 0.131 | 0.000 |
| 39 | ikvu | 0.066 | 0.001 | imup | 0.074 | 0.002 |
| 40 | imno | 0.140 | 0.001 | imus | 0.087 | 0.001 |
