# Supplementary material for: Difficulty in artificial word learning impacts targeted memory reactivation and its underlying neural signatures
Source: eLife. 2024 Nov 4;12:RP90930. doi: 10.7554/eLife.90930 (PMC11534334; doi:10.7554/eLife.90930)
Supplement: Supplementary file 3. — Data are means ± SEM. PP, phonotactic probability. P-values of statistical comparisons between groups by using unpaired t-tests. Note, no significant group differences, but a trend of significance for pre-sleep memory test of c-criterion values of the high-PP condition. [file elife-90930-supp3.docx]

**Supplementary table S3** Gender, age and performance rates

|  | High-PP cued (*n* = 11) | Low-PP cued (*n* = 11) | *t* | *P* |
| --- | --- | --- | --- | --- |
| Female (#) | 10 | 7 | - | - |
| Age (years) | 21.91 ± 0.33 | 22.00 ± 0.56 | -0.14 | 0.89 |
| Pre-sleep memory test | | | | |
| High-PP (d') | 2.11 ± 0.16 | 2.03 ± 0.26 | 0.27 | 0.79 |
| Low-PP (d') | 1.58 ± 0.16 | 1.72 ± 0.23 | -0.53 | 0.60 |
| High-PP (c-criterion) | 0.30 ± 0.09 | 0.57 ± 0.11 | -1.86 | 0.08 |
| Low-PP (c-criterion) | 0.34 ± 0.09 | 0.60 ± 0.14 | -1.60 | 0.13 |
| Psychomotor vigilance task before post-sleep memory test | | | | |
| Reaction time (ms) | 319.97 ± 8.99 | 333.63 ± 10.19 | -1.01 | 0.33 |

Data are means ± SEM. PP, phonotactic probability. *P*-values of statistical comparisons between groups by using unpaired *t*-tests. Note, no significant group differences, but a trend of significance for pre-sleep memory test of c-criterion values of the high-PP condition.
