# Supplementary material for: Difficulty in artificial word learning impacts targeted memory reactivation and its underlying neural signatures
Source: eLife. 2024 Nov 4;12:RP90930. doi: 10.7554/eLife.90930 (PMC11534334; doi:10.7554/eLife.90930)
Supplement: Supplementary file 4. — Data are means ± SEM. P-values (uncorrected for multiple comparisons) of statistical comparisons between groups by using paired t-tests against 0. [file elife-90930-supp4.docx]

**Supplementary table S4** Statistics of SW density time-bin analyses from -0.5 to 3s

| Time-bins (s) | SW density (%) | *t* | *P* |
| --- | --- | --- | --- |
| -0.5 to 0s | 0.21 ± 2.10 | 0.10 | 0.92 |
| 0 to 0.5s | 49.73 ± 14.32 | 3.47 | 0.002 |
| 0.5 to 1s | 83.44 ± 14.97 | 5.57 | <0.001 |
| 1 to 1.5s | 19.77 ± 10.26 | 1.93 | 0.07 |
| 1.5 to 2s | 40.33 ± 10.67 | 3.78 | 0.001 |
| 2 to 2.5s | 5.98 ± 6.37 | 0.94 | 0.36 |
| 2.5 to 3s | 4.81 ± 3.08 | 1.56 | 0.13 |
| Data are means ± SEM. *P*-values (uncorrected for multiple comparisons) of statistical comparisons between groups by using paired *t*-tests against 0. | | | |
