# Supplementary material for: Difficulty in artificial word learning impacts targeted memory reactivation and its underlying neural signatures
Source: eLife. 2024 Nov 4;12:RP90930. doi: 10.7554/eLife.90930 (PMC11534334; doi:10.7554/eLife.90930)
Supplement: Supplementary file 5. — Data are means ± SEM. N1, N2: Non-REM sleep stages N1, N2 and N3; REM, rapid-eye movement sleep; WASO, wake after sleep onset; SW, slow wave; PP, phonotactic probability. P-values of statistical comparisons between groups by using unpaired t-tests. Note, no significant group differences, but a trend of significance for REM sleep parameters. [file elife-90930-supp5.docx]

| **Supplementary table S5** Sleep and reactivation parameter | | | | |  |
| --- | --- | --- | --- | --- | --- |
|  | High-PP cued (*n* = 11) | Low-PP cued (*n* = 11) | *t* | *P* |  |
| Duration (min) |  |  |  | |  |
| N1 | 91.55 ± 20.02 | 65.64 ± 18.77 | 0.94 | 0.36 | |
| N2 | 181.64 ± 11.61 | 200.55 ± 14.5 | -1.02 | 0.32 | |
| N3 | 119.96 ± 15.11 | 99.46 ± 12.11 | 1.06 | 0.30 | |
| REM | 57.91 ± 13.30 | 88.86 ± 9.87 | -1.87 | 0.08 | |
| WASO | 16.96 ± 10.18 | 5.55 ± 1.19 | 1.11 | 0.29 | |
| Duration (%) |  |  |  |  | |
| N1 | 19.44 ± 4.18 | 13.98 ± 3.89 | 0.96 | 0.35 | |
| N2 | 38.85 ± 2.56 | 43.77 ± 3.30 | -1.18 | 0.25 | |
| N3 | 25.54 ± 3.16 | 21.55 ± 2.52 | 0.99 | 0.34 | |
| REM | 12.40 ± 2.82 | 19.46 ± 2.20 | -1.98 | 0.06 | |
| WASO | 3.71 ± 2.27 | 1.23 ± 0.27 | 0.28 | 0.29 | |
| Number of reactivations | | | | |  |
| N2 | 61.00 ± 22.22 | 93.18 ± 24.62 | -0.97 | 0.34 | |
| N3 | 266.18 ± 53.68 | 231.73 ± 34.43 | 0.54 | 0.60 | |
| Slow wave detection | | | | | |
| SW density (#/3s) | 1.87 ± 0.09 | 1.99 ± 0.13 | -0.76 | 0.46 | |
| SW amplitude (µV) | 134.33 ± 14.28 | 107.78 ± 9.96 | 1.53 | 0.14 | |
|  | | | | |  |

Data are means ± SEM. N1, N2: Non-REM sleep stages N1, N2 and N3; REM, rapid-eye movement sleep; WASO, wake after sleep onset; SW, slow wave; PP, phonotactic probability. *P*-values of statistical comparisons between groups by using unpaired *t*-tests. Note, no significant group differences, but a trend of significance for REM sleep parameters.
