# Supplementary material for: Difficulty in artificial word learning impacts targeted memory reactivation and its underlying neural signatures
Source: eLife. 2024 Nov 4;12:RP90930. doi: 10.7554/eLife.90930 (PMC11534334; doi:10.7554/eLife.90930)
Supplement: Supplementary file 6. — Data are means ± SEM. PP, phonotactic probability; SW, slow wave. General density: sleep spindle density during the time window of 0–6 s after stimulus onset in number per trial. SW-coupled: sleep spindles coupled to SW up-states divided by the total number of detected sleep spindles during the time window of interest (0–6 s after stimulus onset) in percent. P-values of statistical comparisons between groups by using unpaired t-tests. [file elife-90930-supp6.docx]

**Supplementary table S6** Density parameters of detected sleep spindles

|  | High-PP cued (*n* = 11) | Low-PP cued (*n* = 11) | *t* | *P* |
| --- | --- | --- | --- | --- |
| Detected sleep spindles | | | | |
| General density (#/trial) | 2.25 ± 0.03 | 2.18 ± 0.11 | 0.60 | 0.56 |
| SW-coupled (%) | 38.10 ± 1.19 | 37.51 ± 2.32 | 0.22 | 0.83 |

Data are means ± SEM. PP, phonotactic probability; SW, slow wave. General density: sleep spindle density during the time window of 0 to 6s after stimulus onset in number per trial. SW-coupled: sleep spindles coupled to SW up-states divided by the total number of detected sleep spindles during the time window of interest (0 to 6s after stimulus onset) in percent. *P*-values of statistical comparisons between groups by using unpaired *t*-tests.
